# Applying the Consolidated Framework for Implementation Research to Identify Implementation Determinants for the Integrated District Evidence-to-Action Program, Mozambique

**DOI:** 10.9745/GHSP-D-21-00714

**Published:** 2022-09-15

**Authors:** Celso Inguane, Caroline Soi, Sarah Gimbel, Nélia Manaca, Isaías Ramiro, Florência Floriano, Georgina de Castro, Orvalho Augusto, Stélio Tembe, James Pfeiffer, Quinhas Fernandes, Kenneth Sherr

**Affiliations:** a Department of Global Health, University of Washington, Seattle, WA, USA.; b Department of Family and Child Nursing, University of Washington, Seattle, WA, USA.; c Health Alliance International, Beira and Chimoio, Mozambique.; d Comité para a Saúde de Moçambique, Maputo City, Mozambique.; e Department of Community Health, Faculty of Medicine, Universidade Eduardo Mondlane, Maputo City, Mozambique.; f National Directorate of Public Health, Ministry of Health, Maputo City, Mozambique.; g Health Alliance International, Seattle, WA, USA.; h Department of Epidemiology, University of Washington, Seattle, WA, USA.; i Department of Industrial & Systems Engineering, University of Washington, Seattle, WA, USA.

## Abstract

**Introduction:**

The Integrated District Evidence-to-Action program is an audit and feedback intervention introduced in 2017 in Manica and Sofala provinces, Mozambique, to reduce mortality in children younger than 5 years. We describe barriers and facilitators to early-stage effectiveness of that intervention.

**Method:**

We embedded the Consolidated Framework for Implementation Research (CFIR) into an extended case study design to inform sampling, data collection, analysis, and interpretation. We collected data in 4 districts in Manica and Sofala Provinces in November 2018. Data collection included document review, 22 in-depth individual interviews, and 2 focus group discussions (FGDs) with 19 provincial, district, and facility managers and nurses. Most participants (70.2%) were nurses and facility managers and the majority were women (87.8%). We audio-recorded all but 2 interviews and FGDs and conducted a consensus-based iterative analysis.

**Results:**

Facilitators of effective intervention implementation included: implementation of the core intervention components of audit and feedback meetings, supportive supervision and mentorship, and small grants as originally planned; positive pressure from district managers and study nurses on health facility staff to strive for excellence; and easy access to knowledge and information about the intervention. Implementation barriers were the intervention’s lack of compatibility in not addressing the scarcity of human and financial resources and inadequate infrastructures for maternal and child health services at district and facility levels and; the intervention’s lack of adaptability in having little flexibility in the design and decision making about the use of intervention funds and data collection tools.

**Discussion:**

Our comprehensive and systematic use of the CFIR within an extended case study design generated granular evidence on CFIR’s contribution to implementation science efforts to describe determinants of early-stage intervention implementation. It also provided baseline findings to assess subsequent implementation phases, considering similarities and differences in barriers and facilitators across study districts and facilities. Sharing preliminary findings with stakeholders promoted timely decision making about intervention implementation.

## Abstract (precis)

We combined the Consolidated Framework for Implementation Research with an extended case study and flexible dissemination strategy. This approach enabled the identification of facilitators and barriers that demonstrated implementation science potential to inform timely adjustments to the implementation of an audit and feedback intervention across multiple sites in Mozambique.

## Teaser

**Key Findings:**

- Our comprehensive and systematic use of the Consolidated Framework for Implementation Research (CFIR), embedded in an extended case study approach, demonstrates how to use the framework to inform data collection and analysis and how to use the case study approach to guide the interpretation of findings to help advance implementation of evidence-informed health programs.
- The Integrated District Evidence-to-Action core intervention components—audit and feedback meetings, supportive supervision and mentorship, and intervention small grants—offered a relative advantage over other similar maternal and child health interventions and routine interventions implemented to reduce mortality in children younger than 5 years.
- These findings illustrate factors that implementers can address to inform mid-program course modifications to implementation of an audit and feedback intervention in the study setting.

**Key Implications:**

- Sharing with stakeholders preliminary, granular, and context-specific findings that reflect the experiences and perspectives of intervention implementers, immediately after data collection and analysis, improves the likelihood of timely decision making about implementation.
- This approach compensates for the gap between data availability and usage, given the longer timeline for publication of study findings.

Resumo em português no final do artigo.

## INTRODUCTION

Worldwide gains in reducing under-5 mortality, due to substantial investments made in low- and middle-income countries (LMICs) such as Mozambique,^1^ have been slowed by persistently high neonatal mortality.^2^ To address this challenge, there is a global consensus to focus on improving coverage and quality of evidence-based interventions delivered at and around the time of birth.^3^^,^^4^ In Mozambique, the implementation of such interventions is facilitated by relatively high utilization of maternal and child health (MCH) services at the primary health care level^5^ and by existing clinical practice guidelines that can be leveraged to increase access to these interventions at scale. A case in point is the Integrated District Evidence-to-Action program to improve maternal, newborn, and child health (IDEAs), a multifaceted modified audit and feedback intervention that has been implemented in Mozambique since the second quarter of 2017. The precursor to the IDEAs program strengthened the health care system and improved coverage of MCH interventions in Mozambique and other African countries between 2009 and 2015.^6^ Improvements documented for the first phase of the precursor intervention are consistent with those demonstrated for other audit and feedback interventions in high-income settings.^7^^–^^9^ Yet the literature lacks evidence on how best to scale up audit and feedback interventions, especially in LMICs.

The IDEAs program was implemented through an iterative 3-step process repeated every 6 months that included: (1) a health systems readiness assessment; (2) district-level audit and feedback meetings where MCH managers from all public sector health facilities reviewed performance and developed action plans to address facility performance gaps; and (3) direct health facility support, through supervision and mentorship coupled with financial assistance to implement facility action plans. The program was implemented in 154 primary health care facilities across 12 districts of Manica and Sofala provinces, Central Mozambique,^6^ reaching nearly 60% of the population of those provinces. Due to resource constraints, the targeted funding and supervision visits focused on 2 low-performing and 1 high-performing facility in each 6-month cycle (and rotated each cycle). Facility performance was assessed during audit and feedback meetings at the end of each 6-month intervention cycle. Performance was based on reaching the targets in select MCH indicators that were national priorities in Mozambique. High-performing facilities were selected to improve staff performance at low-performing facilities, by serving as references for best practices. The intervention was led by MCH managers at the provincial, district, and health facility levels, with technical and logistical support from Health Alliance International (HAI), an international nongovernmental organization (NGO) affiliated with the University of Washington’s Department of Global Health.

IDEAs implementation was an iterative 3-step process: health systems readiness assessment, district-level audit and feedback meetings, and facility-level supportive supervision and mentorship.

The implementation and evaluation of the IDEAs program were guided by a mixed methods approach (quantitative and qualitative), following the Reach, Effectiveness, Adoption, Implementation, and Maintenance (RE-AIM) model.^10^ The Consolidated Framework for Implementation Research (CFIR)^11^ was embedded in the RE-AIM model to generate qualitative evidence to inform national scale up. The CFIR is an established implementation science meta-framework that originally included 39 relatively well-defined constructs organized around 5 conceptual domains: (1) intervention characteristics, (2) outer setting, (3) inner setting, (4) characteristics of individuals, and (5) implementation process.^11^^–^^13^ It is assumed that these domains interact to influence implementation processes and outcomes.^11^^–^^14^ The CFIR can be used systematically in 1 or more intervention phases or across all intervention phases. Yet, to date, the CFIR has mostly been used in the post-implementation phase, to guide data analysis; and some studies have provided the rationale and methods for selecting and using constructs, whereas others have not.^14^^,^^15^ Using the CFIR in this fashion limits its analytical contribution to implementation science efforts to understand determinants of intervention implementation.

We used the CFIR (1) holistically—to guide data collection, analysis, and interpretation; and (2) systematically—by providing our rationale for construct and subconstruct selection, highlighting those with influence on successful implementation. We report on barriers and facilitators to early-stage effectiveness of implementation of the IDEAs program in 4 districts of Manica and Sofala provinces, Central Mozambique.

We used the CFIR to report on barriers and facilitators to early-stage effectiveness of IDEAs in 4 districts in Central Mozambique.

## METHODS

### Study Design and Setting

We used an extended case study design that combined case-oriented and variable-oriented approaches, in which the CFIR was embedded. The case-oriented approach focused on describing the specificities of each case (district) using the CFIR constructs and subconstructs. The variable-oriented approach focused on exploring the similarities and differences across the cases with reference to each construct or subconstruct (“variable”). The CFIR informed the development of data collection tools and the approach to analysis, while the extended case study approach^16^^–^^18^ guided the interpretation of findings.

We collected data in 4 districts of Mozambique (Báruè and Gondola in Manica Province, and Beira and Búzi in Sofala Province), in November 2018, immediately after the first year of implementing the IDEAs program. Mozambique’s national health system is organized into 4 levels of health care attention (primary, secondary, tertiary, and quaternary) with corresponding geographic levels of management (rural or urban, city or district, province, and central). The district, which in some cases is also a municipality, is the most basic level of administrative and financial management of Mozambique’s health system, instead of the health facility. For this reason, the district served as the IDEAs program’s basic level of intervention.

### Participant Selection

The sampling plan consisted of multiple stages. First, we randomly selected 4 of the 12 intervention districts, 2 in each of the 2 provinces where the intervention was being implemented. Second, we purposively selected participants for in-depth individual interviews (IDIs) and focus group discussions (FGDs). We selected 2 provincial health managers (MCH supervisor and head of planning and cooperation) in each of the 2 provinces, 2 district health managers (health director and MCH supervisor) in each of the 4 districts, and 12 MCH nurses—1 from each of the 3 facilities receiving direct intervention support in each of the 4 districts in the semester leading up to data collection. We selected 1 MCH manager from each of the 12 above-mentioned health facilities to participate in FGDs, regardless of having received direct intervention support.

All participants were eligible for the IDI or FGD if they had in-depth knowledge of the intervention. Provincial and district managers’ in-depth knowledge was defined as having participated in intervention activities for at least 12 months at the time of IDI or FGD. Health facility managers’ and nurses’ in-depth knowledge was defined as having attended at least 2 audit and feedback meetings before study participation. At all levels, higher-ranking managers were prioritized for participation but were replaced by lower-ranking ones if they were unavailable at the time of study participation. Each health facility sent only 1 manager and 1 nurse to the district meeting, so we interviewed the 1 present at that meeting if they were eligible. IDIs with provincial managers provided an overview of implementation in the province, while FGDs provided district perspectives. IDIs with district managers and FGDs with MCH managers from facilities that received direct intervention support aimed to provide in-depth understanding of intervention implementation in each district.

All IDIs and FGDs were conducted in person and included 41 participants (22 from IDIs and 19 from 2 FGDs). Most participants were nurses (n=15, 36.6%), MCH facility managers (n=14, 34.1%), and women (87.8%) (Table 1). We reviewed documents that described intervention plans, implementation reports, and presentations made during audit and feedback meetings, and we audio-recorded 88% of the included IDIs and FGDs (n=22/24). We conducted 2 FGDs with MCH facility managers.

**TABLE 1.** Participants in Study on Integrated District Evidence-to-Action Program to Reduce Under-5 Mortality in 4 Districts, Mozambique

**Participants**	**IDI**	**FGD**	**Total** **No. (%)**	**Women** **No. (%)**
Provincial managers	4	0	4 (9.8)	2 (50.0)
District managers	8	0	8 (19.5)	5 (62.5)
MCH facility managers	0	14	14 (34.1)	14 (100)
Nurses	10	5	15 (36.6)	15 (100)
**Total**	22	19	41 (100)	36 (87.8)

Abbreviations: FGD, focus group discussion; IDI, in-depth individual interview; MCH, maternal and child health.

We did not interview MCH nurses from 2 health facilities, because 1 was unavailable for interviewing and another was not eligible because she was new to the intervention. Participants in 2 IDIs and 1 FGD did not consent to audio-recording. We removed 2 FGDs from analysis because of protocol violations: 1 was conducted with MCH managers from facilities not included in the intervention, and the other was conducted with participants who had already completed IDIs.

Interviews were conducted after study participants gave their written informed consent. They gave consent separately for documenting the interviews using notes and audio-recording. Audio-recordings and notes were assigned individual alphanumeric codes that protected the identification of each key informant. Before preparing this article, study investigators obtained key informants’ feedback on preliminary study findings developed from IDI and FGD notes. To protect the identity of study participants, we replaced district names with codes (A, B, C, D) and used general participant categories, such as “provincial or district manager” (without mentioning the province or district), and “nurse,” or “nurse manager” (without specifying the health facility).

### Data Collection

Two teams of 3 interviewers with academic training in the social sciences, humanities, or public health research and 3 or more years of research experience in MCH, supervised by 2 study investigators, conducted the assessment over 3 weeks in November 2018. Before conducting data collection, teams received 5 days of training on procedures in human subjects research and data collection and management, and data collection and supervision instruments were pretested. Team composition, supervision, and debriefing meetings were used to improve data quality and analysis. IDIs and FGDs were conducted in Portuguese. For each IDI, 1 team member conducted the interview while a second member documented it through field notes and audio-recording, if participants consented to the procedure. FGDs were run by all 4 team members: 1 facilitator, 2 notetakers, and an observer (study investigator). Study investigators conducted supportive supervision by observing at least 1 interview led by each interviewer, where they observed the quality of rapport and interview techniques, time spent during the interview, and how participant anonymity and data confidentiality were ensured. At the end of each supervised interview, the study investigator provided feedback to the interviewer and, if needed, discussed with them how to improve forthcoming interviews.

At the end of each day, the study investigator led a team debriefing meeting to share supervision feedback, review findings, and conduct preliminary data analysis. The team also identified potential protocol violations or adverse events and other challenges and planned for the following day of data collection.

### Data Analysis

As a first step to assess the validity of our findings,^19^ at the end of the data collection period, the teams presented preliminary district reports to district managers and the HAI implementation team in Mozambique. Each report contained key findings organized around the core intervention characteristics and the barriers and facilitators to successfully implementing the intervention, which participants had identified.

In-depth data analysis was consensus-based and iterative, following a mixed deductive and inductive approach, using a predefined codebook containing CFIR constructs and subconstructs organized around the 5 original conceptual domains (https://cfirguide.org/tools/tools-and-templates/). Data analysis was conducted using ATLAS.ti 8.4, where data was stored and entries into the codebook, including definitions of conceptual entities (domains, constructs, and subconstructs), were made. After audio-recordings were transcribed, for 2 months between May and August 2019, 3 study investigators with experience in qualitative research methods and CFIR application analyzed data from IDI and FGD transcripts and notes and from documents containing intervention details.

To ensure consistency in the analysis, the 3 investigators standardized analysis procedures in a daylong workshop. Thereafter, 2 investigators independently analyzed the same data (transcripts, notes, and documents) for each study facility and district and met daily to resolve discrepancies in coding and to jointly produce district-specific memos. Data analysis started with 12 constructs that study investigators had prioritized based on their research experience using the CFIR and working in the intervention geographic area (deductive approach). These constructs included “linkages among intervention components,” a construct that was not originally in the CFIR but that was based on the investigators’ experience using the CFIR. Study investigators were open to adding other CFIR constructs and subconstructs that they had not anticipated but that emerged during data analysis (inductive approach). This iterative approach allowed for adding 4 unanticipated CFIR constructs (Table 2). When consensus was not reached, a third investigator (tiebreaker) with experience using the CFIR resolved differences. Then, 1 of the 2 original investigators entered the agreed-upon codes and ratings in ATLAS.ti, after which the 2 original investigators met to write district memos.

**TABLE 2.** CFIR Constructs and Subconstructs Used to Analyze Study on Integrated District Evidence-to-Action Program to Reduce Under-5 Mortality in 4 Districts, Mozambique

**CFIR Domains**	**Constructs and Subconstructs Used in Final Analysis**
Intervention characteristics	Intervention source Relative advantage Adaptability
Outer setting	Peer pressure External policy and incentives
Inner setting	Structural characteristics^a^ Networks and communications^a^ Compatibility Relative priority Access to knowledge and information (subconstruct of readiness for implementation)^a^
Characteristics of individuals	Knowledge and beliefs about the intervention Self-efficacy
Process	Planning Innovation participants (subconstruct of engaging)^a^ Executing Linkages among intervention components^b^

Abbreviation: CFIR, Consolidated Framework for Implementation Research.

a Construct/subconstruct that had not been prioritized before analysis but was added in the final analysis.

b Construct is not original to CFIR but is based on the investigators’ experience using CFIR.

Ratings followed an approach developed by Damschroder et al., which defines the valence and strength of each CFIR construct or subconstruct.^11^^,^^12^ Valence denotes the positive or negative influence of the construct or subconstruct on implementation,^11^^,^^12^ which we defined as a facilitator or a barrier. Strength indicates (1) the level of emphasis, which is determined by the descriptive language participants used; (2) whether concrete examples were provided; and (3) the level of participant agreement on language and/or examples.^11^^,^^12^ Positive valence is indicated by +, and its strength can be weak (+1) or strong (+2), whereas negative valence is indicated by -, and its strength can be weak (-1) or strong (-2).^11^ Valence of constructs and subconstructs can also be neutral (0) if they have unclear directional influence, and their influence can be mixed (X) if the positive and negative influences cancel each other out.^11^^,^^12^ A construct or subconstruct was deemed significant in a district if at least 2 participants mentioned it and was considered important to the evaluation if it was mentioned in at least 2 districts.

We also adapted and expanded the CFIR analysis process by using an extended case study approach, including a case-oriented and a variable-oriented approach.^16^^–^^18^^,^^20^ Using the case-oriented approach, the investigators coded and wrote memos for each IDI or FGD; using the variable-oriented approach, investigators selected constructs and subconstructs and wrote memos by site (district). Site-specific memos included construct and subconstruct ratings, with a narrative justifying the ratings and providing details (e.g., participant quotes) as appropriate. We then presented district-specific reports, in lay language in Portuguese, to each district to (1) assess validity of our findings through participant feedback, (2) promote evidence-based decisions about adjustments to the intervention, and (3) fulfill an ethical obligation of returning research findings to study participants. To prepare this article, we synthesized the key findings from district reports, highlighting differences and commonalities in barriers and facilitators, wherever relevant, and noting where data was not enough to warrant a conclusion on whether certain themes worked as facilitators or barriers.

We adapted and expanded the CFIR analysis process by using an extended case study approach, including a case-oriented and a variable-oriented approach.

### Ethics Approval

The study was approved by the institutional review board of the University of Washington (IRB#STUDY00003926), Mozambique’s National Bioethics Committee for Health (*Comité Nacional de Bioética para a Saúde*-CNBS-IRB00002657), and the Ministry of Health, after endorsement from Manica and Sofala Provincial Health Directorates.

## RESULTS

### Core Intervention Components

Study participants identified 3 core intervention components, namely audit and feedback meetings, supportive supervision and mentorship, and financial support grants, which study participants identified as: discussion round, technical support, and purchase of materials. They named the intervention after its financial component and the intervention donor, by calling the intervention the Doris Duke Subagreement.

Audit and feedback meetings occur every 6 months at the district capital to assess health facility performance, categorize health facilities, and select those that will receive technical support and mentorship.

*And funds are disbursed annually for data discussions, twice a year. So, what happens is data discussions to assess performance. Depending on the performance of the health facility, we categorize health facility in those who perform well with reference to indicators and those who are worse. So, 3 health facilities are selected to receive technical support after the discussion. So, 2 low performing and 1 better performing. So, we can conduct supervision and provide technical support to them*. —District manager, November 2018

Participants described how during supportive supervision and mentorship visits to health facilities, district managers identify technical and material needs of health facilities and provide technical support.

*So, we do supervision and technical support, during which we see what is going well and what is not. If the issue is only lack of knowledge, we provide technical support. However, during those visits we see, as I said earlier, the issue of supplies. If work is not being performed because there are no gloves; if work is not being performed because there is no thermometer.* —District manager, November 2018

A study participant aptly summarized other participants’ descriptions of how financial support grants were used to purchase medical supplies for health facilities and to support audit and feedback meetings at the district.

*[F]unds are disbursed annually for data discussions, twice a year [;] then, with the subagreement funds from HAI we purchase those supplies and give them to health facilities. —*District manager, November 2018

### Facilitators to Successful Implementation

We defined successful implementation of the intervention as close as possible to how the intervention had been described in the intervention protocol. Facilitators to successfully implementing the IDEAs intervention were widespread across 4 of the 5 CFIR domains (intervention characteristics, outer setting, inner setting, and process), while barriers were only captured under the 2 domains of intervention characteristics and inner setting (Table 3). In the Supplement, we present the full list of significant constructs and subconstructs and their influence on intervention implementation.

**TABLE 3.** Facilitators and Barriers to Successfully Implementing Study on Integrated District Evidence-to-Action Program to Reduce Under-5 Mortality in 4 Districts, Mozambique

**CFIR Domains**	**Facilitators**	**Barriers**
Intervention characteristics	Advantage of core intervention components (audit and feedback meetings, supervision and mentoring, and financial support) compared to routine and similar MCH interventions (relative advantage)	Limited flexibility of intervention design, and decision making about data collection tools and use of intervention small grants (adaptability)
Outer setting	Positive pressure district managers and NGO staff exerted over health facility staff (external policy and incentives)	---
Inner setting	Historically established professional networks and communications among NGO and government staff and managers at provincial and subprovincial levels (networks and communication)	
	Relative priority given to MCH at national and subnational levels of Mozambique’s health system (implementation climate)	Intervention did not adequately address nurse shortages and training needs, health facility infrastructure, and district transportation needs—compatibility (implementation climate)
	Access to knowledge and information about the intervention (readiness for implementation)	
Process	Approach to implementing intervention components as originally planned (executing)	---

Abbreviations: MCH, maternal and child health; NGO, nongovernmental organization.

The main facilitators to successfully implementing the IDEAs intervention were the relative advantage that the intervention’s core components brought to implementation and executing the approach to implementation as originally planned, the positive pressure that district managers and HAI study nurses exerted on health facility staff performance, and the access to knowledge and information about the intervention. Facilitators that had limited influence on effective intervention implementation (not represented in all districts) included the influence of professional networks and communication between health facility staff and managers, district managers, and provincial NGO staff and the priority given to reducing maternal and child mortality at various levels of Mozambique’s health system.

#### Advantages of Core Intervention Components and Implementation Approach

Study participants thought that the intervention’s core components gave it a relative advantage compared to routine activities and other interventions that focused on MCH at the district level. Specifically, participants noted that financial assistance enabled district directorates to fund health facility supervision visits, purchase equipment and other materials, and perform maintenance and repairs at facilities. Participants also indicated that during supervision and mentorship visits and audit and feedback meetings, nurses learned new competencies from district managers and fellow nurses that helped nurses standardize work procedures and interpret routine health information. This helped improve MCH indicators and the quality of services provided at their facilities.

Study participants thought the core components of the intervention gave it relative advantage compared to routine activities and other district-level MCH interventions.

Participants also thought the intervention was being executed as described in district subagreements, which helped participants become more conversant with intervention components and activities over time. They reported additional advantages such as the biannual audit and feedback meetings funded by the intervention helping keep nurses abreast of developments in Ministry of Health guidelines and health service delivery strategies. They also reported that those meetings provided district and facility MCH managers opportunities to analyze data and discuss facility action plans as a group in a mentoring environment. Therefore, participants thought that this multipronged approach helped nurses think creatively. In some cases, the approach resulted in tangible facility-level management improvements, such as holding weekly data audit meetings at the health facility to help improve data quality and better prepare for district audit and feedback meetings. Finally, during an FGD, nurses noted that the intervention had a rigorous theoretical framework and pragmatic implementation approach that addressed the individual professional needs of MCH nurses and helped them improve their data management skills, evaluate their own performance, and proactively address identified gaps.

*In my case, I see this HAI project as helping me know my work better. This project helps in practice as well as in theory. It is rigorous, it is demanding at the theoretical and at the practical level, and it helps you in what is wrong with you. Compared to other projects, HAI helps us open our minds so we can understand where we are, where we are going and where we want to get to. For instance, during data audit meetings, we open our minds. We see where we are at, where we are going and where we want to get to.* —Nurse FGD, Sofala Province, November 2018

#### Positive Pressure and Access to Knowledge and Information

Health facility nurses expressed that pressure from district managers and HAI nurses was an important positive influence because it “pushed nurses to strive for excellence” in data registration, reporting, and service delivery. This positive influence was also manifested by district managers using existing Mozambican public service policy (*Estatuto Geral dos Funcionários e Agentes do Estado*, 2009) to enforce reporting deadlines anticipated in the national health information system, whenever needed. Participants also mentioned that HAI nurses motivated them, by following up with health facility teams on implementation of facility action plans, helping those teams brainstorm solutions and prioritize actions, and giving prizes to health facilities that met the goals they had set in action plans.

Audit and feedback meetings and supervision visits also helped successfully implement the IDEAs program by providing access to knowledge and information related to the intervention and other MCH topics. During audit and feedback meetings, nurses exchanged ideas and experiences about what they had learned in nursing school and learned about what other facilities were doing to meet intervention objectives. This was supported by mentoring and supervision visits, during which district managers observed nurses’ work, identified challenges, monitored implementation of health facility action plans, and mentored nurses on how to overcome challenges in implementing those plans. Supervision and mentoring were effective because whenever nurses needed additional technical assistance outside of the supervision visits, they could obtain such assistance through a phone call to district managers.

Supervision and mentoring were effective because nurses could obtain assistance through phone calls to district managers whenever they needed.

#### Networks and Communication and Maternal and Child Health as a National Priority

Access to knowledge and information in the IDEAs intervention operated in a larger context of effective working relationships and communication practices involving district and health facility teams, provincial health managers, and study staff from the implementing NGO (HAI). As study participants noted, open and consistent communication worked well, especially for troubleshooting, timely referrals of emergency cases to district hospitals, and allocation and use of subagreement funds. As a provincial manager noted, these communication practices were facilitated by relationships built during the precursor to the IDEAs program (2009–2015), which often helped to overcome challenges related to using and reporting on subagreement funds.

*Who implements, who decides what needs to be done, is the district first. So, we as provincial directorate and the partners (HAI) help in that process and the same [happens] at the coordination level. When it comes to planning, we are together. So, there is no difference. One hand washes the other and two hands wash the face. This is what has been happening since the first [phase of the] project that we implemented. From the level of planning, through discussion, when it comes to matrices, to discussions, everything is coordinated. The level of coordination is extremely high.* —Provincial manager, November 2018

Although the intervention focuses on MCH, which is a priority at the various levels of Mozambique’s health system, this had a strong positive influence in only 1 district, where district and facility managers and staff from all programs actively participated in intervention activities and helped maintain resources acquired through the small intervention grants.

### Barriers to Successful Implementation

Barriers to successful implementation of the IDEAs program across all 4 districts were related to compatibility and adaptability. The intervention lacked compatibility in not addressing resource constraints at district and facility levels and it lacked adaptability in having too little flexibility in its design and decision making about using data collection tools and intervention funds.

Barriers to successful implementation of IDEAs across all 4 districts were related to lack of compatibility and adaptability.

#### Lack of Compatibility

Participants thought the IDEAs intervention was incompatible with local needs because it did not adequately address MCH nurse shortages, MCH nurses’ training needs, health facility infrastructure, or district transportation needs. Most in-service MCH training provided by the government and other initiatives besides the intervention was short term, and some health facilities had only 1 MCH nurse or none, in which case a general nurse provided MCH services. If the only facility nurse went to the district capital for trainings or meetings, which often occurred, she would be replaced by colleagues from other sectors who were unprepared to manage obstetric emergencies and made data entry mistakes that nurses had to answer for in district audit and feedback meetings. Yet, technical assistance during intervention-sponsored supervision and mentorship visits did not extend to the supporting, non-MCH colleagues. Study participants also noted that health facilities had inadequate MCH infrastructure, including in some cases, all 5 MCH sectors sharing the same physical space. In other cases, electricity infrastructure was weak for equipment such as incubators for newborns, and districts had few vehicles to serve over 10 facilities, some of which were very far from each other. Consequently, it becomes difficult to provide adequate newborn care and referrals for emergency cases or transport medical supplies and samples across facilities.

#### Lack of Adaptability

To these areas of incompatibility, study participants added several adaptability barriers related to the intervention’s thematic and age focus, decision making about the use of data entry tools, and use of subagreement funds. Participants found the intervention design to be restrictive, as it focused on a certain age group (neonates), did not include all health facility sectors during supervision visits, and did not cover all facilities in the district in every intervention cycle of 6 months each year. They added that the matrix used to assess facility performance metrics was constantly changing, which caused confusion, and their suggestions to improve the matrix were dismissed by their superiors under the argument that the matrix could be changed only at the central level. Finally, they noted that subagreement amounts were insufficient and rules for their use were restrictive and counterproductive.

*What we feel this time is the limitation on the number of participants in the meetings. For instance, we have 17 health facilities [in the district] and […] we used to divide the group so that we had about 60 people in each group. When we started this project, because of the funding mechanism, we were told that each room needs to have up to 30 people. So, this is a limitation. [Now] the performance level of the discussion went down ... As we know maternal and child health is not an island; it’s not isolated; so, all other programs [need to] participate in the implementation [of this intervention]. That’s why I say that it was challenging when they imposed a limit on the number of people [who could] attend the meeting.* —District manager, November 2018

## DISCUSSION

This study leveraged the CFIR to identify facilitators and barriers to successfully implementing the early stage of the IDEAs program, a multifaceted and modified audit and feedback intervention that has been implemented in 12 districts of Manica and Sofala provinces in Mozambique since 2017. We shared preliminary findings with stakeholders from the 4 districts included in the evaluation to inform timely decision making about intervention implementation and provided findings that also served as a baseline for subsequent assessments during and after the implementation of the IDEAs program.

The main facilitator of implementation was implementing the core intervention components as originally planned. Those components included district audit and feedback meetings, supportive supervision and mentorship to health facilities and their staff, and small grants provided to districts to facilitate the implementation of health facility action plans. District managers and study nurses who exerted positive pressure over health facility managers and nurses further facilitated implementation, aided by the access to information about the intervention that district and health facility managers and nurses enjoyed. This was enhanced by the good working relationships and communication practices that provincial, district, and health facility teams and HAI staff had developed during implementation of the precursor to the IDEAs program in 2009–2015. The prioritization of reducing maternal and child mortality in Mozambique also played a minor role in facilitating the successful implementation of the IDEAs program. Other studies that used the CFIR systematically in LMICs, including in Mozambique, also identified relative advantage^21^^,^^22^ and relative priority^13^^,^^23^ as facilitators.

Barriers were less prominent and included the intervention’s inability to address the human, infrastructural, logistical, and funding resource needs of districts and facilities; and the intervention’s limited flexibility in design and decision making about data collection tools and use of financial resources. The importance of compatibility as a barrier to successfully implementing interventions in LMICs has been highlighted elsewhere.^23^

Our evaluation was consistent with other studies conducted in Mozambique that could not assess whether the involvement of community members and other stakeholders^13^ and the availability of resources^13^^,^^24^ in local health systems influenced successful implementation of interventions.

Our study supplements the relatively small proportion of empirical studies that have used the CFIR systematically and comprehensively. We used the CFIR during implementation, whereas most studies have used it after implementation.^14^^,^^15^ We also used the CFIR for multiple methodological purposes (to guide our approach to data collection, analysis, and interpretation of findings), similar to most other studies in LMICs,^15^ while embedding the CFIR’s analytical approach into an extended case study design,^16^^–^^18^ which helps intervention stakeholders use findings to make decisions about intervention implementation in 1 site (district) or across different sites included in the intervention.

### Limitations

The perspectives of some participants were excluded from this analysis because we removed data from 2 FGDs that violated the study protocol. This limitation does not seem likely to reduce the validity of our findings, which we confirmed through stakeholder feedback on preliminary findings. The likelihood of information bias that is common when obtaining self-reported data was reduced through triangulation of data collection methods (i.e., individual interviews, FGDs, and document review), and by using a robust analysis approach (i.e., sharing findings with stakeholders and combining consensus-based analysis with a tiebreaker system). The very detection of protocol violations reflects the effectiveness of procedures that the study team employed to ensure ethical integrity and methodological rigor. To preserve analytical consistency, our analysis did not include the full scope of improvements in the CFIR which were made after we had developed the study protocol. An instance is the recent addition to the CFIR of a sixth domain—characteristics of systems—and its associated 6 constructs, which is part of efforts to improve the CFIR’s applicability to LMICs^15^ including Mozambique.^13^^,^^21^^,^^25^ The inclusion of such improvements in future studies will be important since the CFIR has mostly been used in high-income health systems.

## CONCLUSIONS

Our evaluation of the first year of implementing the IDEAs program, a multifaceted and modified audit and feedback intervention in Mozambique, suggested that its successful implementation was facilitated by the approach to implementing the core intervention components as originally planned. This was enhanced by supportive communication and mentoring practices partially grounded on historically established relationships involving NGO and local health system actors developed during implementation of the precursor to the IDEAs program in Mozambique. However, challenges in the intervention design of incorporating provisions to address human resources, infrastructural, and financial needs of districts and health facilities could threaten the successful implementation of the subsequent phases of the intervention.

## Supplementary Material

GHSP-D-21-00714-supplement.pdf

## Translation

**Em Português**

**Aplicação do Quadro Conceptual Consolidado para Pesquisa de Implementação à Identificação dos Determinantes do Programa Distrital Integrado de Evidência-para-acção em Moçambique**

**Principais Resultados**

- O nosso uso abrangente e sistemático do Quadro Conceptual Consolidado para Pesquisa de Implementação (CFIR) inserido num estudo de caso estendido, demonstra como usar o quadro para informar a recolha e análise de dados e, como usar o estudo de caso para guiar a intepretação de resultados, para melhorar a implementação de programas de saúde informados por evidência.
- Os principais componentes do Programa Distrital Integrado de Evidência-para-acção, nomeadamente, a reuniões de avaliação de dados (*audit&feedback*), supervisão e apoio técnico e, apoio financeiro, conferiram vantagens relativamente a intervenções de rotina e outras intervenções similares de saúde materno-infantil implementadas para reduzir a mortalidade em menores de 5 anos.
- Estes resultados ilustram os factores que implementadores podem abordar para informar modificações durante a implementação de uma estratégia assente na avaliação de dados (*audit&feedback intervention*) no local do estudo.

**Principais Implicações**

- A partilha atempada (imediatamente após a recolha de dados) de resultados preliminares, detalhados e contextuais com os principais interessados, que reflectem as experiencias e perspectivas dos implementadores da intervenção, melhora a possibilidade de tomada de decisões atempadas sobre a implementação.
- Esta abordagem reduz o tempo entre a disponibilidade dos resultados e o seu uso, dada a demora na publicação dos resultados de pesquisa cientifica.

**Resumo**

**Introdução:** O Programa Distrital Integrado de Evidência-para-acção (IDEAs) para redução da mortalidade neonatal, é uma intervenção que inclui a revisão de dados (*audit&feedback*), implementada desde 2017, nas províncias de Manica e Sofala, Moçambique, para reduzir a mortalidade em crianças menores de 5 anos. Apresentamos aqui os facilitadores e as barreiras para as fases iniciais de implementação desta intervenção.

**Métodos:** Inserimos o Quadro Conceptual Consolidado para Pesquisa de Implementação (CFIR) num desenho de estudo de caso (*extended case study design*) para orientar a amostragem e, recolha, análise e interpretação de dados. Colhemos dados em 4 distritos das províncias de Manica e Sofala em novembro de 2018. A recolha de dados incluiu a revisão documental, 22 ‘entrevistas’ individuais em profundidade e 2 discussões em grupo focal (DGF) com 19 enfermeiras de SMI de unidades sanitárias e gestores de Saúde Materno-Infantil (SMI) de nível provincial, distrital e de unidades sanitárias. A maioria dos participantes (70.2%) foram enfermeiras e gestoras das unidades sanitárias e mulheres (87.8%). Entretanto, não gravamos duas entrevistas e 2 DGF. Realizamos uma análise iterativa baseada no consenso entre os analistas.

**Resultados:** Facilitou a implementação eficaz intervenção (a) a implementação das componentes centrais da intervenção – reuniões de revisão de dados, supervisão e apoio técnico e, apoio financeiro – conforme originalmente planificado; (b) a pressão positiva que os gestores distritais e as enfermeiras do estudo exerceram sobre o pessoal das unidades sanitárias para que atingissem excelência no trabalho e; (c) o acesso facilitado à informação sobre a intervenção. Constituíram barreiras à implementação (i) a incompatibilidade da intervenção, por não responder à escassez de recursos humanos, financeiros e infraestruturas adequadas para o os serviços de SMI nos distritos e unidades sanitárias e; (ii) a falta de adaptabilidade da intervenção, expressa pela reduzida flexibilidade no desenho e tomada de decisão sobre os instrumentos de recolha de dados e uso de fundos alocados pela intervenção.

**Discussão:** O nosso uso holístico e sistemático do CFIR inserido num desenho de estudo de caso gerou evidência detalhada sobre a contribuição deste quadro conceptual para os esforços das ciências de implementação em descrever os determinantes das fases iniciais de implementação de uma intervenção. Igualmente, forneceu dados de linha de base que podem ser usados para avaliar fases subsequentes da implementação da intervenção, tomando em conta similaridades e diferenças entre barreiras e facilitadores entre os distritos e unidades sanitárias. A partilha dos resultados preliminares com os principais interessados promoveu a tomada de decisão atempada sobre a implementação da intervenção.

**Peer Reviewed**
