# Supplementary material for: Applying the Consolidated Framework for Implementation Research to Identify Implementation Determinants for the Integrated District Evidence-to-Action Program, Mozambique
Source: Glob Health Sci Pract. 2022 Sep 15;10(Suppl 1):e2100714. doi: 10.9745/GHSP-D-21-00714 (PMC9476477; doi:10.9745/GHSP-D-21-00714)
Supplement: GHSP-D-21-00714-supplement.pdf [file GHSP-D-21-00714-supplement.pdf]

Supplement to: Inguane C, Soi C, Gimbel S, et al. Applying the Consolidated Framework for Implementation Research to identify implementation determinants for the Integrated District Evidence to Action Program, Mozambique. *Glob Health Sci Pract*. 2022;10(Suppl 1):e2100714. https://doi.org/10.9745/GHSP-D-21-00714

Supplement. Ratings of Constructs and Subconstructs by District

CFIR Domains, Constructs, and Subconstructs	District A	District B	District C	District D
**Intervention characteristics**				
Intervention source	0	0	0	0
Relative advantage	+2	+2	+2	+2
Adaptability	-2	-2	-2	-1
**Outer setting**				
Peer pressure	+2	Missing	Missing	+1
External policy and incentives	+2	+2	+2	+2
**Inner setting**				
Structural characteristics	-1	Missing	Missing	-2
Networks and communications	+2	+1	+2	+2
Implementation climate				
Compatibility	-1	-2	-2	-2
Relative priority	+2	+1	+1	+1
Readiness for implementation				
Access to knowledge and information	+2	+2	+2	+2
**Process**				
Planning	Missing	+2	+1	0
Engaging				
Innovation participants	-1	+2	+2	+2
Executing	+2	+2	+2	+2
Linkages among intervention components[a]	+2	+2	0	+1
**Characteristics of individuals**				
Knowledge and beliefs about the intervention	0	+1	+1	+2
Self-efficacy	+2	+2	+1	x

Abbreviation: CFIR, Consolidated Framework for Implementation Research.
Note: + and – indicate valence, i.e., a positive or negative influence of a construct or subconstruct on implementation. Strength of influence can be weak (1), strong (2), neutral (0) if the direction of construct/subconstruct influence is unclear (negative/positive), or mixed (X) if the positive and negative influence cancel each other out.

[a] Construct is not original to CFIR but is based on the investigators' experience using the CFIR.
